# Configurational asymmetry in vernier offset detection

**DOI:** 10.2478/v10053-008-0077-1

**Published:** 2010-10-06

**Authors:** A. K. M. Rezaul Karim, Haruyuki Kojima

**Affiliations:** 1 Department of Psychology, University of Dhaka, Bangladesh; 2 Graduate School of Human and Socio-environment Studies, Kanazawa University, Japan

**Keywords:** vernier acuity, offset direction, orientation cue, configuration, training, response bias, asymmetry, neural preference, cortical plasticity

## Abstract

Two psychophysical experiments were conducted at the horizontal and vertical orientations respectively, demonstrating substantial main effect of configuration, but no effect of offset direction on vernier acuity. In Experiment 1, a pair of horizontal bars were arranged side by side with a large gap between them. The observers were, on average, significantly better at discriminating a vertical offset if the right-hand bar was below the left-hand bar than vice versa, regardless of which bar they experienced as displaced and which as constant. A similar asymmetry was evident in Experiment 2 where observers judged horizontal offset for a pair of vertically oriented bars, where one was placed above the other. In this case average performance was better if the upper bar was on the right of the lower bar rather than on its left. There were large individual variations in the asymmetrical trend, but the effect could not be explained by subjective response bias. Furthermore, vernier acuity improved significantly and the asymmetry decreased more or less as a function of training. The average asymmetrical trend was consistent across training days and across two orientations, which indicates that the processing of line vernier stimuli is possibly configuration-specific in the cardinal orientation.

## INTRODUCTION

Visual acuity plays an important role in humans’ daily lives. For example, it helps them to read and write everyday documents, organize items, drive cars and/or follow directions. It is crucial for skilled performance in spatially complex tasks such as surgical procedures. One popular measure of visual acuity is vernier acuity. *Vernier acuity* is the capacity to perceive a spatially offset visual stimulus such as detecting whether two thin lines are aligned or misaligned. Scientists have been working on understanding how vernier acuity is accomplished for several decades. Their conclusions thus far have been that orientation tuning of cortical neurons (Campbell & Kulikowski, 1966; Phillips & Wilson, 1984) provides an important source of information by which the visual system accomplishes this job (cf. Sullivan, Oatley, & Sutherland, 1972; Watt, Morgan, & Ward, 1983; Waugh, Levi, & Carney, 1993; Wilson, 1986). The receptive fields of V1 neurons, with different orientation preferences and slightly different receptive field positions, are clearly able to discriminate between a straight vernier and an offset one (Wilson, 1986), between an offset to left and an offset to the right (Poggio, Fahle, & Edelman, 1992), or between an offset up and an offset down.

**Figure 1.**
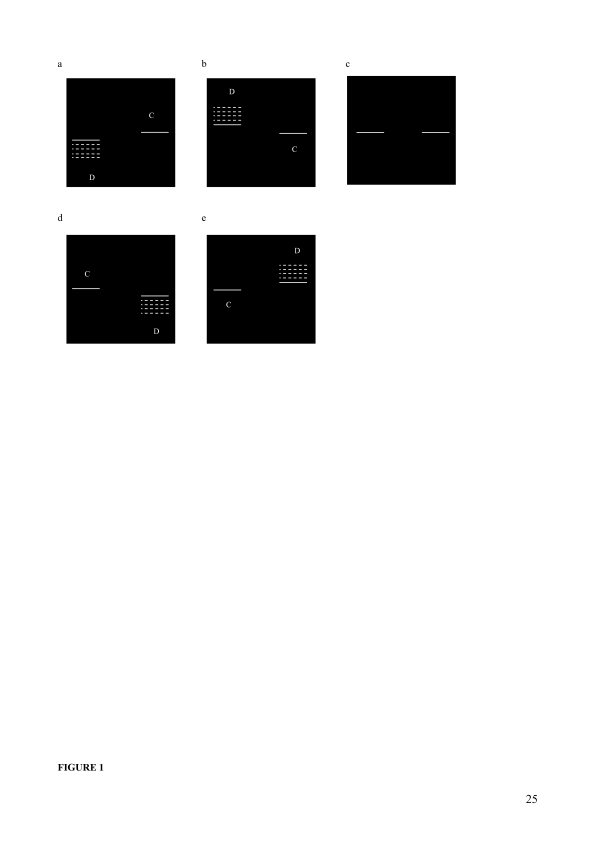
Schematic of the vernier stimuli used in Experiment 1. (a) Stimuli with downward offset of the left bar. (b) Stimuli with upward offset of the left bar. (c) Stimulus with null-offset. (d) Stimuli with downward offset of the right bar. (e) Stimuli with upward offset of the right bar. Vernier separation is defined as the horizontal distance between the endpoints of the left and right bars, and vernier offset is defined in arcseconds as the vertical distance between the two bars. The dotted lines indicate the approximate positions of the displaced bar with different offset sizes (“D” and “C” are used here and subsequently where necessary to represent the “Displaced” and “Constant” bars, respectively).

One factor that may contribute to vernier offset discrimination is the orientation cue created by the feature offset (Andrews, Butcher, & Buckley, 1973; Fahle, 1991; Sullivan et al., 1972; Watt, 1984; Watt et al., 1983). The orientation cue created by the offset in either direction (e.g., up or down) is a unique spatial property of vernier stimuli and cannot be considered as the stimulus orientation itself. When vernier features (light bars) are arranged such that the left feature is above the right one both the orientation cue and configuration in space become different to when the arrangement is reversed, with all other parameters being identical (Figure 1). Scientists have repeatedly demonstrated that vernier offset in either direction is discriminated better at a cardinal rather than an oblique orientation (e.g., Saarinen & Levi, 1995b; Skrandies, Jedynak, & Fahle, 2001). This perceptual asymmetry has a neural basis insofar as more V1 neurons are devoted to the cardinal than to the oblique orientation (Coppola, White, Fitzpatrick, & Purves, 1998; Furmanski & Engel, 2000; Li, Peterson, & Freeman, 2003), but they can also be altered or modified by visual experience (Sengpiel, Stawinski, & Bonhoeffer, 1999; White, Bosking, & Fitzpatrick, 2001). However, it remains unknown whether there is perceptual asymmetry in the way the left and right vernier features (at 0º orientation), or upper and lower vernier features (at 90º orientation), are displaced from each other. It is assumed that there may be some asymmetry in orientation cue perception produced by the feature offset, or by the luminance edges of the vernier stimuli, or an asymmetry in configuration perception structured by its spatial frame. If this assumption proves to be true this leads to another possibility; namely, that learning shapes the asymmetry (cf. Adams, Graf, & Ernst, 2004; Champion & Adams, 2007). Examining whether spatial orientation perception has an asymmetric nature in a simple line vernier configuration is therefore worthwhile.

Two experiments were, therefore, carried out using the line vernier stimuli, at 0º and 90º orientations respectively, to explore offset directional, configurational and training effects on vernier performance. The term *direction* here refers to the displaced feature’s offset to up or down (0º oriented vernier) and to left or right (90º oriented vernier) from the constant feature, whereas *configuration* represents the whole stimulus frame, comprised of the features’ relative spatial positions, luminance edges, and the feature offset, such as a configuration with the left feature up and the right feature down or vice versa. The experiments demonstrated substantial main effects for configuration and training, but no effect for offset direction on vernier acuity.

## EXPERIMENT 1: DETECTING VERNIER OFFSET AT 0º ORIENTATION

### Method

#### Observers

Twelve paid adults (2 graduate and 10 undergraduate students) of normal, or corrected to normal, vision were used in the experiment. The observers did not know about the purpose of the experiment and did not have any history of psycho-physiological or neurological illness.

#### Stimuli and apparatus

Horizontal line vernier stimuli, either aligned or misaligned, were generated using Borland C++ Builder 6. Each stimulus was comprised of two light bars, one of which was displaced up (–) and down (+) at right angles to the other, constant, bar. The constant bar was always in the same vertical position across its horizontal locations (right, left). The stimuli were white against a black background, with a feature separation of 22.5 arcmin (Figure 1). The width and length of each feature were 0.5 and 15 arcmin, respectively. The offset sizes of the misaligned stimuli were ± 30, ± 60, ± 90, ± 120, and ± 150 arcsec. A luminance meter (TOPCON BM-3) was used to measure the luminance of the stimulus and background. The Michelson contrast of each stimulus was 0.98 (L_max_ = 90.43 cd/m^2^, L_min_ = 0.81 cd/m^2^). A 21-inch CRT colour monitor (Eizo, FlexScan T962) of 1280 x 1024 pixels and 85 Hz with a high-speed graphic card (3Dlabs Wildcat III 6110) was used to display the stimuli. From a viewing distance of 1.82 m the angular resolution of each pixel was 30 arcsec.

#### Procedures

At the beginning, observers were allowed to practice a few times in order to give them some practical knowledge of how to respond using the keyboard. Following the method of constant stimuli, the stimuli were presented in two different sessions. In one session, five possible vernier stimuli of the downward offset (+) and five aligned (null-offset) verniers were randomly presented in the central visual field. Similarly, in another session five possible stimuli of the upward offset (–) and five aligned verniers were presented. The stimulus duration was 100 ms. The response-stimulus interval (an interval between the onset of a response in the present trial and the onset of a stimulus in the following trial) was 1000 ms. The order of the two sessions was counterbalanced between the observers, and between the training days for each observer. Each session included 80 repetitions of each stimulus covering 800 trials in total (400 offset and 400 aligned verniers). Observers in a dark room were asked to view the stimuli binocularly using a chin and forehead rest from the distance stated above. They were asked to press a key (“F” or “J”) in order to indicate whether the bars were aligned or misaligned. Each incorrect response (responding to an aligned vernier as misaligned or vice versa) of the observers’ was followed by an auditory feedback. The two response keys were counterbalanced between the observers. There was no additional fixation point, in order to avoid unwanted positional cues available from that point (Waugh & Levi, 1993). However, observers were instructed, in advance, to pay attention to the gap between the bars (the centre of the display).

The experiment ran for 6 days. Half of the observers experienced the misaligned stimuli with the left bar displacement (Figure 1a, b) and the remaining half experienced the right bar displacement (Figure 1d, e), and the null-offset vernier (Figure 1c) was experienced by all in each experimental session. However, observers were not informed about which bar they experienced as displaced and which as constant (a situation of spatial uncertainty).

#### Data processing and statistical analysis

In each session, the proportion of correct offset detection at each offset and the proportion of false detection (i.e., detection of a null-offset vernier as an offset one) were calculated for individual observers. The proportion of false detections was used to determine subjective response biases. If an observer had response bias towards a particular vernier configuration it would be accompanied by higher proportion of false detections in that session compared to the opposite configurational session. In other words, the distribution of false detections would not be uniform in the upward offset and downward offset (which form two comparable configurations) sessions. So, a very simple technique was used to calculate the relative response bias of each observer on each training day. That is, each observer’s upward or downward response bias was determined using Equation 1 (cf. Nicholls, Hughes, Mattingley, & Bradshaw, 2004; Nicholls, Mattingley, Bradshaw, & Krins, 2003).

(1) $$\text{Response Bias = }(F_{u}-F_{d})\times 100$$

Where, *F*_u_ and *F*_d_ represent the proportions of false detections, on each day, in the upward and downward offset sessions respectively. Then, response biases for each observer were averaged over the training days. The average response bias could, therefore, range from –100 to +100, with negative and positive values reflecting downward and upward biases respectively. A score approaching zero indicates no response bias towards or against any configuration.

**Figure 2.**
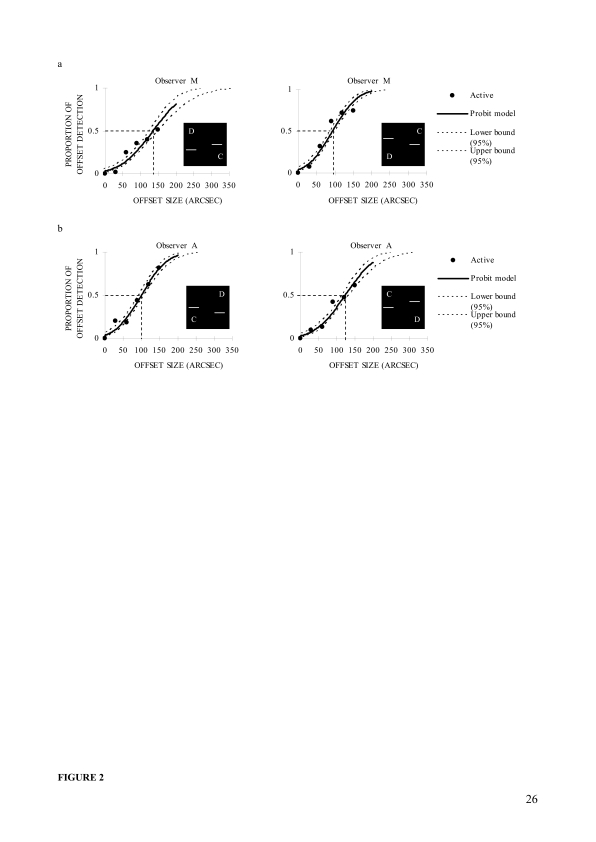
Psychometric functions of two typical observers in the first training day in Experiment 1. (a) Psychometric functions of observer M for the downward (left panel) and upward (right panel) displacements of the left vernier bar. (b) Psychometric functions of observer A for the downward (left panel) and upward (right panel) displacements of the right vernier bar.

The response bias scores were analyzed in a series of one-sample *t*-tests. After exclusion of the subjective biased responses on a day-by-day basis, where necessary, the data (proportions of correct offset detections) in the upward and downward offset sessions were separately fitted using the probit model (cf. Fahle, Edelman, & Poggio, 1995; Mussap & Levi, 1997) using XLSTAT (Addinosoft USA). Then, using the Yes/No paradigm offset detection threshold was calculated, in each session, at 50% correct detection of the vernier misalignment. Figure 2 displays the psychometric functions of two typical observers, one on the left and another on the right bar displacements, on the first day of training. In order to understand the effects of different factors, group threshold data was analyzed in repeated measures ANOVA tests (followed by the post-hoc LSD test where appropriate), and each observer’s threshold data was analyzed in a matched sample *t*-test. For repeated measures ANOVA the Greenhouse-Geisser correction was applied if a factor had more than two levels. This corrects for possible violation of the sphericity assumption in repeated measures data (Greenhouse & Geisser, 1959; Jennings, 1987; Vasey & Thayer, 1987). However, two observers (O2 and O12) having correct response proportions substantially higher for smaller offset sizes than for larger ones and/or showing higher false detection than correct detection, even at larger offsets (i.e., response by guessing), were considered unreliable and hence excluded from the analysis.

### Results and discussion

#### Response bias

**Figure 3.**
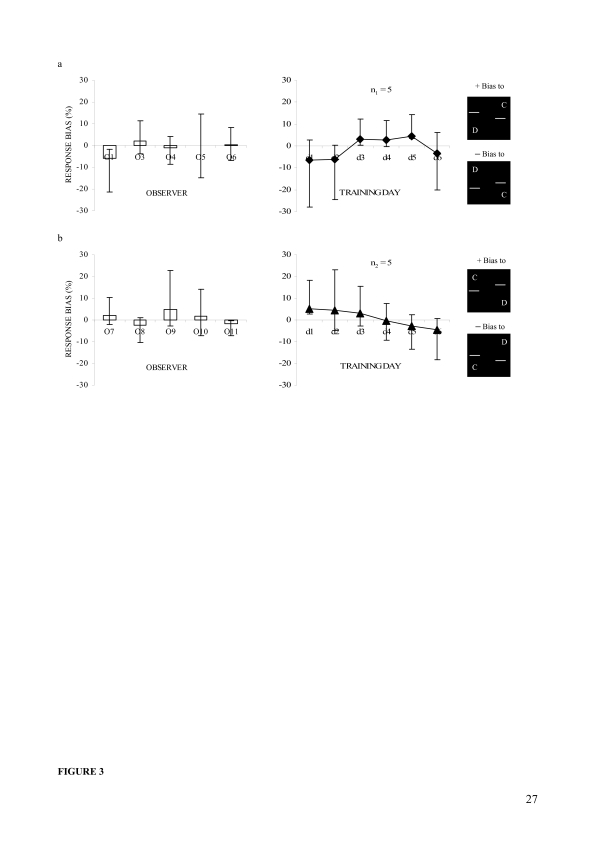
Response biases (Mean ± ***CI***s) in Experiment 1. (a) Percent biases in the left bar displacement, left panel: subjective biases of five observers, right panel: mean daily biases of the group. (b) Percent biases in the right bar displacement, left panel: subjective biases of other five observers, right panel: mean daily biases of the group. The positive (+) and negative (–) values indicate biases to upward and downward offsets, respectively. Error bars reflect 95% confidence intervals (***CI***s) of the mean differences.

Figure 3 shows mean subjective response biases calculated over the training days (left panels) and mean daily response biases for the two observer groups, one in the left bar (section a, right panel) and another in the right bar (section b, right panel) displacement scenario. The left panels indicate that mean bias scores were upward for O3, O6, O7, O9, and O10 (+ scores) and downward for the other observers (– scores). When subjected to a series of one-sample *t*-tests the bias score was not found to be significantly different from zero for any observer. It was not even significant for O1 and O11 who may show some bias in the figure. The daily response bias data, as shown in the right panels, were also analyzed in a series of one-sample *t*-tests, which revealed that these scores were not significantly different from zero for either observer group. This was even true for d5 in the left bar and d1 in the right bar displacement scenario. The right panels also indicate that the distribution of the mean daily bias scores for both groups were random across the training days rather than indicating that the magnitude of bias (whatever the degree) reduced with training.

#### Offset direction, configuration, and training effects

##### Overall effects

**Figure 4.**
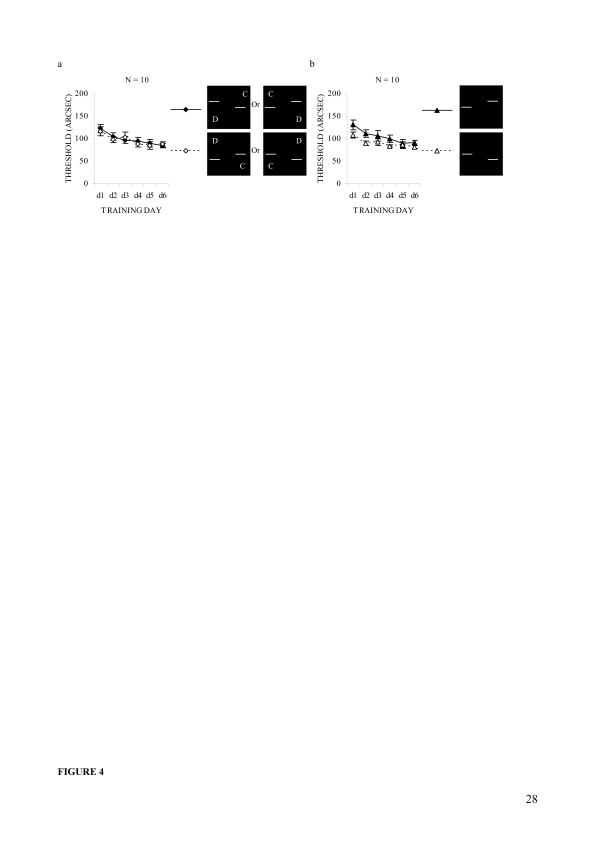
Average performances by offset direction, configuration, and training in Experiment 1. (a) Offset direction-wise daily mean thresholds of all observers irrespective of which bar they were experiencing as displaced. (b) Configuration-wise daily mean thresholds of all observers irrespective of which bar they were experiencing as displaced. Error bars reflect standard errors (***SE***s) of the means.

Average offset detection thresholds, for all the observers, are plotted by offset direction (Figure 4a) and by configuration (Figure 4b) using training as a common factor. To see the overall effects of different factors the observers’ threshold data was first analyzed in two-way repeated measures ANOVA tests, with offset direction and training as within-subjects factors. The sphericity assumption was violated for training factor and interaction, so the Greenhouse-Geisser correction was applied. It revealed that the main effect of training was significant, Greenhouse-Geisser corrected *F*(2.93, 26.37) = 8.3, ε = .586, *p* < .001; but the effects of offset direction and its interaction with training were not. The data was then analyzed using the same statistical procedures, considering configuration and training, as within-subjects factors. It was found that the main effects of configuration and training were significant, *F*(1, 9) = 9.3, *p* = .014 for configuration; Greenhouse-Geisser corrected *F*(2.93, 26.37) = 8.3, ε = .586, *p* < .001 for training; but the effect of their interaction were not. Further analysis of the training effect was done using the post hoc LSD test, which revealed that the threshold was significantly lower on the second day of training (*M* = 101.3, *SE* = 6.2, *p* = .003) than on the first (*M* = 119.9, *SE* = 6.7). This improvement was maintained on the third (*M* = 99.9, *SE* = 9.3, *p* = .004), fourth (*M* = 91.9, *SE* = 4.7, *p* = .001), fifth (*M* = 87.8, *SE* = 8.2, *p* = .002) and sixth (*M* = 86.3, *SE* = 4.3, *p* < .001) days.

The configurational differences were also examined for both pre- and post-training. To do so, the first day’s training was considered as pre-training and sixth (last) day’s training as the post-training. The pre-training mean threshold difference between the two configurations was around 22 arcsec (*SE* = 10.5), the difference was reduced to about 8 arcsec (*SE* = 4.2) in the post-training (Figure 4b). However, matched sample *t*-tests revealed that the pre-training mean difference was fairly large, *t*(9) = 2.1, *p* = .065, and the post-training mean difference was significant, *t*(9) = 2.3, *p* = .044.

##### Individual trends

**Figure 5.**
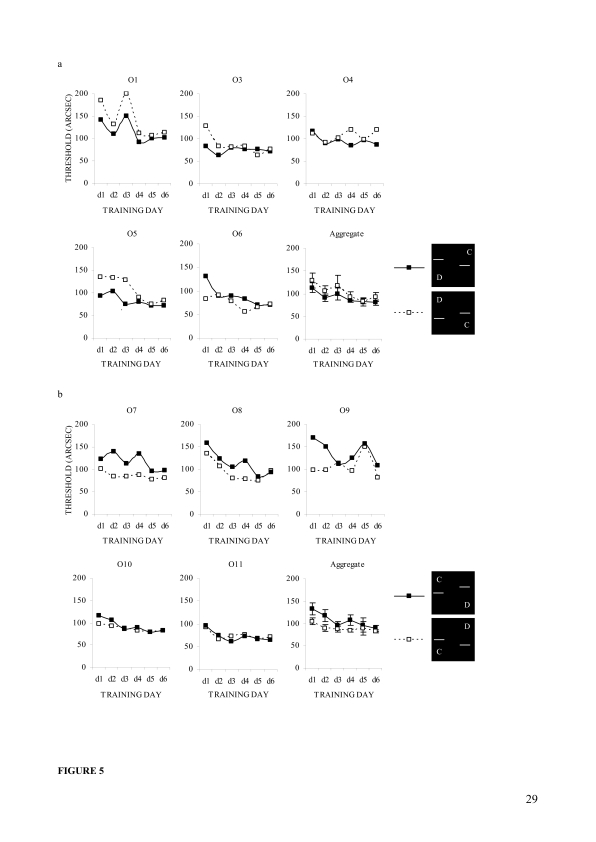
Individual differences in configuration and training effects in Experiment 1. (a) Configuration-wise daily observer thresholds and the corresponding aggregate for five observers in the left bar displacement. (b) Configuration-wise daily observer thresholds and the corresponding aggregate for other five observers in the right bar displacement. Error bars reflect standard errors (***SE***s) of the means.

Figure 5 displays, by configuration, the daily offset detection thre-sholds for individual observers and the corresponding aggregates for the two observer groups that experienced the misaligned stimuli, with the left and right bar displacements respectively. A series of matched sample *t*-tests applied to the data demonstrated that two (O1 and O5) of the five observers that experienced the left bar displacement (Figure 5a) had significantly lower thresholds if the left bar’s offset was upward, *t*(5) = 3.7, *p* = .014 for O1; *t*(5) = 3.1, *p* = .026 for O5. Three (O7, O8, and O9) of the five observers experiencing the right bar displacement (Figure 5b) had significantly lower thresholds if the right bar’s offset was downward, *t*(5) = 4.9, *p* = .004 for O7; *t*(5) = 3.0, *p* = .029 for O8; and *t*(5) = 2.8, *p* = .040 for O9. Other observers in the two groups did not show any asymmetry of this kind, indicating individual differences in the trend. However, line graphs of the aggregated data for the two groups show that average thresholds were lower if the left bar was displaced to up (Figure 5a, last panel) and the right bar was displaced to down (Figure 5b, last panel), compared to the opposite displacements. The differences were fairly large in the right bar displacement, *F*(1, 4) = 6.8, *p* = .060, but not in the left bar displacement. However, the trends are configurationally identical irrespective of which bar the observers experienced as displaced.

To summarize, it was found that vernier threshold depended on vernier configuration and training, but not on offset direction. That is, observers’ average threshold was significantly better in the LU-RD (left feature up vs. right feature down) than in the LD-RU (left feature down vs. right feature up) configuration, irrespective of which bar they experienced as displaced (Figure 4b). This effect was significant for 50% of the observers, but no significant subjective response bias was detected towards or against any configuration. In addition, both the vernier threshold and size of the average asymmetry consistently decreased with training (Figure 4b), though it was still statistically significant in the training course.

## EXPERIMENT 2: DETECTING VERNIER OFFSET AT 90º ORIENTATION

### Method

#### Observers

Twelve naïve and paid adults of normal or corrected to normal vision participated in this experiment.

#### Stimuli and apparatus

Line vernier stimuli, either aligned or misaligned, were used at a 90º orientation with a feature separation of 20 arcmin (figure not shown). The offset sizes of the stimuli, feature length, feature width, and stimulus contrast were all identical to Experiment 1. The apparatus used was also the same.

#### Procedures

The setup and procedures were identical to Experiment 1, and the experiment took 12 days.

#### Data processing and statistical analysis

As in Experiment 1, the leftward or rightward subjective response biases were calculated and analyzed in a series of one-sample *t*-tests. After the subjective biased responses on a day-by-day basis were excluded, where necessary, the data (proportions of correct offset detections) in the leftward and rightward offset sessions were separately fitted by probit model (cf. Fahle et al., 1995; Mussap & Levi, 1997). Then offset detection threshold was calculated, in each session, at 50% correct detection of the vernier misalignment. In order to reduce the effect of presentation order (of the configuration) on any pair of subjective thresholds, the threshold data was averaged on every two successive days of training. Thus, in 12 days of training, six pairs of scores were obtained for each observer. Then inferential analyses of the data was done following the same statistical tools that were used in the first experiment. Two observers (O6 and O12) who showed higher false detection than correct detection, even at larger offsets (i.e., response by guessing), were considered unreliable and hence excluded from the analysis.

### Results and discussion

#### Response bias

**Figure 6.**
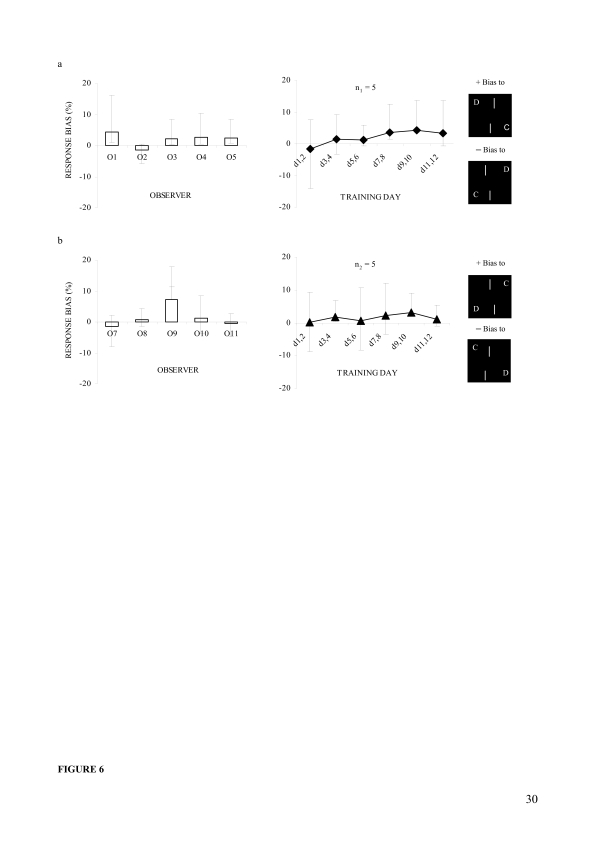
Response biases (Mean ± ***CI***s) in Experiment 2. (a) Percent biases in the upper bar displacement; left panel: subjective biases of five observers, right panel: group mean biases in every two successive days of training. (b) Percent biases in the lower bar displacement, left panel: subjective biases of other five observers, right panel: group mean biases in every two successive days of training. The positive (+) and negative (–) values indicate biases to rightward and leftward offsets, respectively. Error bars reflect 95% confidence intervals (CIs) of the mean differences.

Figure 6 shows mean subjective response biases calculated over the training days (left panels) and mean response biases calculated on every two successive days for the two groups, one in the upper bar (section a, right panel) and another in the lower bar (section b, right panel) displacement scenario. The left panels indicate that the mean bias scores were rightward for O1, O3, O4, O5, O8, O9, and O10 (+ scores) and leftward for the other observers (– scores). When subjected to a series of one sample *t*-tests, the bias score was only found to be significantly different from zero for O9, *t*(11) = 5.0, p < .001. It was not significant even for O1, O3, O4, and O5 who may show some bias in the figure. So, before determining offset detection thresholds, the response bias was excluded, on a day-by-day basis, for O9 only. The response bias data, as shown in the right panels, was also analysed in a series of one-sample *t*-tests, which revealed that these scores were not significantly different from zero for either group. This was true even for d7.d8 and d9.d10 in the upper bar and for d3.d4 and d9.d10 in the lower bar displacement scenario. The right panels also indicate that the distribution of the mean response bias scores for both groups was almost rightward across the training days regardless of which bar was experienced as displaced, and that the magnitude of bias (whatever the degree) did not reduce with training.

#### Offset direction, configuration, and training effects

##### Overall effects

**Figure 7.**
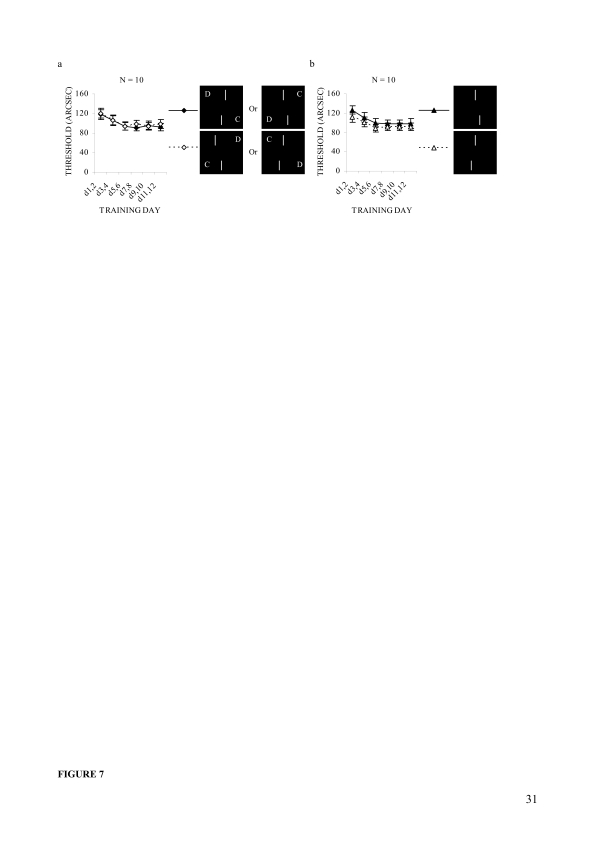
Average performances by offset direction, configuration and training in Experiment 2: (a) Offset direction-wise mean thresholds in every two successive days for all observers irrespective of which bar they were experiencing as displaced. (b) Configuration-wise mean thresholds in every two successive days for all observers irrespective of which bar they were experiencing as displaced. Error bars reflect standard errors (***SE***s) of the means.

Offset detection thresholds averaged over all the observers are plotted by offset direction (Figure 7a) and by configuration (Figure 7b) using training as a common factor. As in Experiment 1, observers’ threshold data was analysed using two-way repeated measures ANOVA tests, first with offset direction and training and then with configuration and training as within-subjects factors. The analysis revealed that the main effect of configuration was nearly significant, *F*(1, 9) = 5.0, *p* = .052, and that of training was significant, Greenhouse-Geisser corrected *F*(1.81, 16.32) = 7.5, ε = .363, *p* = .006. However, the main effect of offset direction and neither of the two-factor interaction effects (Offset direction x Training; Configuration x Training) were significant. A further analysis of the training effect, using the post hoc LSD test, revealed that the third and fourth days’ mean threshold (*M* = 106.1, *SE* = 9.6) was significantly lower than the first and second days’ mean threshold (*M* = 119.2, *SE* = 9.2, *p* = .002). This improvement was maintained at the fifth and sixth (*M* = 94.1, *SE* = 7.6, *p* < .001), seventh and eighth (*M* = 94.7, *SE* = 6.9, *p* < .001), ninth and tenth (*M* = 94.9, *SE* = 7.7, *p* = .002), and eleventh and twelfth (*M* = 95.3, *SE* = 8.2, *p* = .013) days’ follow-ups.

The configurational differences were also examined in both pre- and post-training. To do so, the first and second days’ training were considered as pre-training and the eleventh and twelfth days’ training as the post-training. The pre-training mean threshold difference between the two configurations was about 14 arcsec (*SE* = 6.8), the difference was reduced to about 6.5 arcsec (*SE* = 5.9) in the post-training (see Figure 7b). Matched sample *t*-tests revealed that the pre-training mean difference was fairly large, *t*(9) = 2.1, *p* = .067, but the post-training mean difference was not.

##### Individual trends

**Figure 8.**
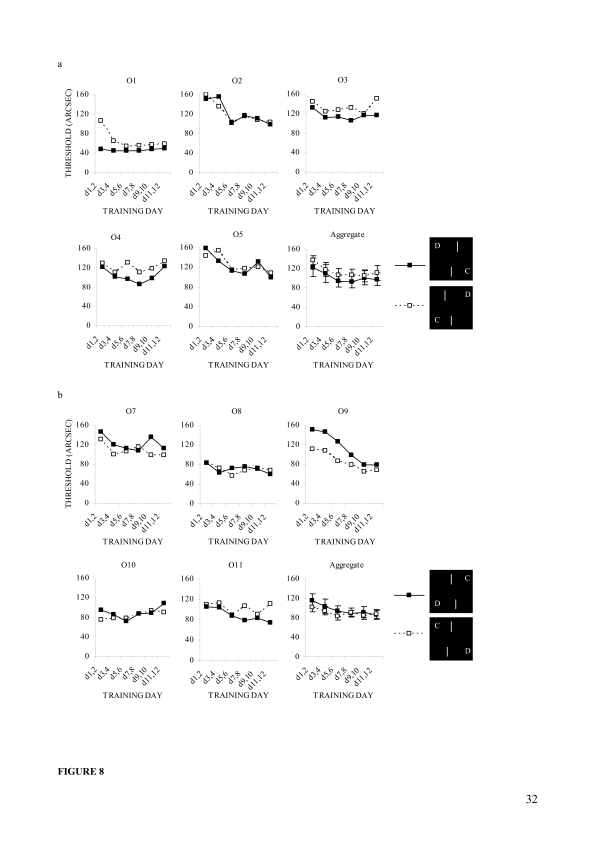
Individual differences in configuration and training effects in Experiment 2. (a) Configuration-wise observer thresholds calculated in every two successive days of training and the corresponding aggregate for five observers in the upper bar displacement. (b) Configuration-wise observer thresholds calculated in every two successive days of training and the corresponding aggregate for other five observers in the lower bar displacement. Error bars reflect standard errors (***SE***s) of the means.

Figure 8 displays, by configuration, the offset detection thresholds, averaged every two successive days of training for individual observers. It also displays the corresponding aggregates for the two groups that experienced the misaligned stimuli with the upper and lower bar displacements respectively. A series of matched sample *t*-tests applied to the data demonstrated that three (O1, O3, and O4) of the five observers experiencing the upper bar displacement (Figure 8a) had significantly lower thresholds if the upper bar’s offset was rightward, *t*(11) = 3.3, *p* = .007 for O1; *t*(11) = 3.7, *p* = .003 for O3; *t*(11) = 4.0, *p* = .002 for O4. On the other hand, two (O7 and O9) of the five observers experienced the lower bar displacement (Figure 8b) as having significantly lower thresholds if the lower bar’s offset was leftward, *t*(11) = 3.0, *p* = .012 for O7; *t*(11) = 5.0, *p* < .001 for O9, while only one observer (O11) showed an opposite trend, *t*(11) = 2.6, *p* = .024. Other observers of the two groups did not show this sort of asymmetry, indicating individual differences in the trend. However, line graphs of the aggregated data for the two groups show that mean thresholds were lower if the upper bar was displaced to right (Figure 8a, last panel) and the lower bar was displaced to left (Figure 8b, last panel) as compared to their counterparts. The differences were fairly large in the upper, *F*(1, 4) = 6.4, *p* = .065, but not in the lower bar displacement. However, the trends are configurationally identical irrespective of which bar the observers experienced as displaced.

To summarize, significant response bias towards a particular vernier configuration was only detected for one observer. This subjective bias, on a day-by-day basis, was excluded before calculating his/her thresholds. The main effect of configuration was found to be nearly significant, but the effect of offset direction on vernier threshold was not found to be significant. That is, observers’ average threshold was marginally better in the UR-LL (upper feature to right vs. lower feature to left) than in the UL-LR (upper feature to left vs. lower feature to right) configuration, irrespective of which bar they experienced as displaced (Figure 7b). At the individual level, the effect was significant for 50% of the observers. Training improved average vernier threshold and reduced the asymmetry substantially.

## GENERAL DISCUSSION

**Figure 9.**
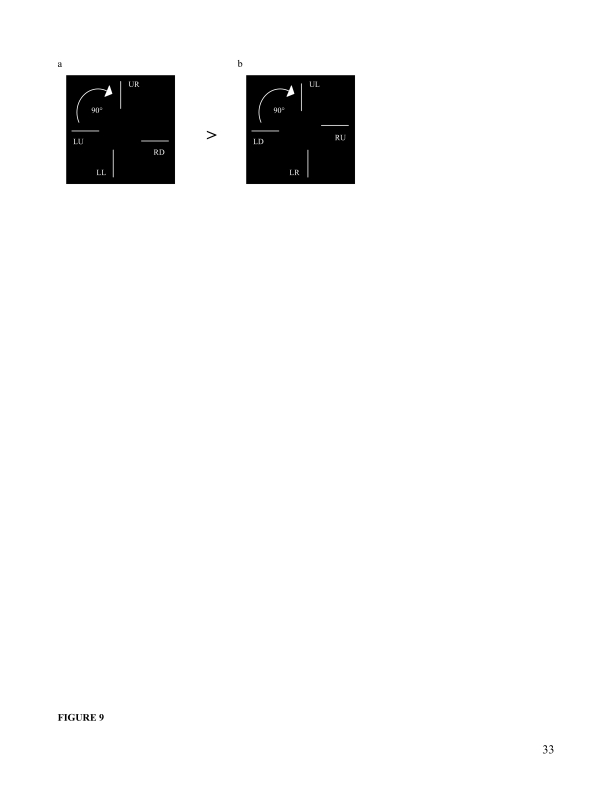
Schematic of the vernier configurations in comparison. (a) 0° and 90° oriented configurations in which average performance was better. (b) 0° and 90° oriented configurations in which average performance was worse.

Two line vernier experiments were conducted at the cardinal orientation on naïve and independent observer groups. In these experiments the main effect of configuration on vernier acuity was found to be significant or nearly significant, but the effect of offset direction was not found to be significant. Specifically, for a pair of horizontal bars arranged side by side with a large spatial gap, in Experiment 1 obser-vers were, on average, significantly better at discriminating a vertical offset if the right-hand bar was below the left-hand bar than vice versa, regardless of which bar they experienced as being displaced. That is, average vernier acuity was finer in the LU-RD than in the LD-RU configuration (Figure 4b), but there was no response bias towards or against any configuration (Figure 3). A similar asymmetry was also evident for horizontal offset detection in Experiment 2, which used a pair of vertically oriented bars; one above the other. In that case, average performance was marginally better in the UR-LL compared to the counter (UL-LR) configuration (Figure 7b), with the exclusion of subjective response bias where necessary (Figure 6). Consistency can be seen in the average findings of the two orientations (Figure 9) if the vernier configurations at horizontal orientation are compared to the corresponding configurations at vertical orientation. That is, a rotation of configuration LU-RD (Figure 9a, horizontal) 90° clockwise refers to configuration UR-LL (Figure 9a, vertical), and similarly a rotation configuration LD-RU (Figure 9b, horizontal) 90°clockwise refers to configuration UL-LR (Figure 9b, vertical).

In addition, in both the orientations the average asymmetrical trend was highly consistent across the training days (Figures 4b, Figure 7b). Though the trend was inconsistent between the observers it was highly consistent within the observers (Figure 5a,b; Figure 8a,b) and this was even true for the only observer (O11) who showed an opposite trend in the second experiment. Thus, the results are reasonable and interesting.

### Training effect, configurational asymmetry, and response bias

This study showed that the mean offset detection threshold improved significantly with training. Once improvement occurred it became highly stable until the end of the training course (Figures 4a,b and 7a,b). The results indicate that the neural reorganization that was necessary for improved performance favourably and consistently occurred throughout the training course in both experiments. The interindividual differences in learning vernier acuity, however, were striking. In Experiment 2, for example, O9 showed a remarkable fall in vernier thresholds during the course of training, whereas O3 and O4 did not show any noticeable improvement (Figure 8a,b). The large individual variation in learning vernier acuities is in agreement with previous studies (Fahle & Edelman, 1993; McKee & Westheimer, 1978; Saarinen & Levi, 1995a). For instance, McKee and Westheimer (1978) reported that after 2000 to 2500 trials the range of the individual decrease in vernier thresholds was from 2 to 70%.

In spite of the significant effect of training on average vernier acuity line graphs show a consistent trend in the configurational asymmetry, to at least some degree, across the training days in two experiments (Figures 4b and 7b). For example, the pre-training average asymmetries in the experiments were around 15 to 20 arcsec, the post-training asymmetries were around 5 to 10 arcsec. These values are numerically small, but have perceptual significance as they fall in hyperacuity level, a fraction of the diameter of a foveal photoreceptor, that the human visual system is able to exhibit (cf. Fahle, 1991, 2004; Harris & Fahle, 1995; Poggio et al., 1992; Westheimer, 1976, 1977). The post-training asymmetries seemed to reduce numerically in both experiments, however; it was still significant in Experiment 1, which only trained observers for 6 days, and non-significant in Experiment 2, which trained observers for 12 days. An inspection of the individual observers’ data indicates that training decreased the asymmetry more or less not only in Experiment 2 (e.g., O1 and O9; Figure 8a,b), but also in Experiment 1 (e.g., O1, O5, O7, O8, and O9; Figure 5a,b). It is, therefore, suggested that there might be configuration-selective neural processing of the line vernier stimuli. And due to plasticity of cortical neurons (Eichenbaum, 2002; Kandel, Schwartz, & Jessell, 2001) this response property might be refined or modified by learning, resulting in less or no asymmetry in the long run of a training course.

An important aspect of this study is that training improved the offset detection threshold, but did not affect response bias in any way. There are two possibilities for this differential effect. First, training did not affect response bias as it was non-significant (i.e., no bias). Second, the response bias and threshold may be associated with the accuracy and precision of the measurement respectively. If a psychophysical system that mediates alignment judgments is assumed the system’s accuracy and precision can be influenced by different factors. As has been shown, unlike the offset detection threshold, the mean response bias scores were distributed randomly across the training days for both the groups in Experiment 1 (Figure 3, right panels), and in Experiment 2 the distribution almost showed a rightward trend regardless of which bar was experienced as displaced (Figure 6, right panels). Thus, the data can be interpreted as indicating that the threshold, which may reflect the system’s precision, depends on the configuration, but the response bias, which may reflect the system’s accuracy, does not.

### Why is this sort of asymmetry?

As discussed above, it can be argued that even if there is no response bias there can be asymmetry. It is unlikely that eye movement contributed to this asymmetry as the stimulus duration was very brief (100 ms) in this study. Nevertheless, if there had been any eye movements it might have no role in vernier acuities (Keesey, 1960), because vernier lines have internal orientation information, and may therefore be less susceptible to orientational or angular noise created by head tilt or eye torsion (cf. Waugh & Levi, 1993). The possibility of visual field asymmetries can also be ruled out because such asymmetries have been evident in peripheral vision only. For example, it has been demonstrated that upper-lower visual field asymmetries are observed at eccentricities larger than about 5° (Portin, Vanni, Virsu, & Hari, 1999). But the present study used offset sizes of 30 to 150 seconds of arc (0.5 to 2.5 minutes of arc). The sizes of feature separation were 22.5 (Experiment 1) and 20 (Experiment 2) minutes of arc both being much less than 5° of arc. Why, then, is there this sort of asymmetry?

As the present results suggest, cortical neurons might have a preference or selectivity for one particular vernier configuration rather than another. Visual response properties are thought to develop in two distinct phases: an experience independent phase in which the basic neural circuits become established and organized into cortical maps, and a subsequent phase of plasticity in which initial circuits are elaborated and refined by experience (Crair, Gillespie, & Stryker, 1998; Hubel & Wiesel, 1963; Katz & Crowley, 2002; Sengpiel & Kind, 2002). The asymmetric or preferential response being reported here cannot be attributed to the first candidate as it is still unknown whether there has been any inborn corresponding asymmetry of neural organizations in the visual cortex. The second candidate explains the asymmetry better because most aspects of spatial vision (e.g., vernier acuity, grating acuity) are quite immature in the human neonate (Skoczenski & Norcia, 1999) and neural organization of the human visual system may be influenced by its early visual input (Freeman, Mitchell, & Millodot, 1972; Freeman & Thibos, 1973; Mitchell, Freeman, Millodot, & Haegerstrom, 1973). Thus, the preference might have developed as a result of early biased learning. The present study also provides a couple of good reasons that could explain the fact in this way. First, 50% of the observers showed significantly better performances in a particular vernier configuration and this figure was highly consistent between experiments. A few of the observers showed this kind of asymmetry to some degree, one showed an opposite trend and several others did not show any asymmetry at all (Figures 5a,b and 8a,b), thus indicating large individual variations in the trend. This may be because our experiential worlds are not necessarily equal for all. The differential visual experiences during the critical period of development may result in individual variability of neural mechanisms that encode the angular positions of visual stimuli (Greene, Frawley, & Swimm, 2000). Second, the degree of configurational asymmetry decreased more or less as a function of training for 70% of those observers, who showed the asymmetry significantly (Figures 5a,b and 8a,b). It can be argued that the asymmetry, which possibly developed through early experience or through evolution, became minimized or decreased during the course of training in our study. However, this idea does not necessarily conflict with the fact that innate asymmetry can also decrease with training, as cortical neurons are plastic (Eichenbaum, 2002; Kandel et al., 2001).

The results of this study are not only interesting but also surprising. This may be because of the complexity of our visual system and the diversity of our visual experiences. Past studies have convincingly shown that top-left lighting preference can be real in visual spatial judgment (e.g., Elias & Robinson, 2005; Mamassian & Goutcher, 2001; Sun & Perona, 1998) though it may not be apparent in human’s conscious awareness and cannot be causally related to any known cortical function. Similarly, the present study adds the information that the visual system may prefer a particular arrangement of light bars (i.e., vernier configuration). Thus, there is a possibility that humans have some anisotropic properties in visual perception. It remains unclear whether this anisotropy is innate or acquired.
